# NET-like Events on Peripheral Blood Smears at Admission: Association with Disease Severity and Systemic Inflammation in Hospitalized COVID-19 Patients

**DOI:** 10.3390/medicina62010153

**Published:** 2026-01-12

**Authors:** Alexy Rosales, Rodrigo Boguen, Felipe Garrido, Francisco Quiñones, José Barros, Fabián Baeza, Josefa Díaz, Salvador Fuentes, Pablo Letelier, Neftalí Guzmán

**Affiliations:** 1 Laboratorio de Investigación en Salud de Precisión, Centro de Investigación en Ciencias de la Salud, Departamento de Procesos Diagnósticos y Evaluación, Facultad de Ciencias de la Salud, Universidad Católica de Temuco, Manuel Montt 56, Temuco 4780000, Chile; arosales@uct.cl (A.R.); rboguen@uct.cl (R.B.); jignaciobarrosp@gmail.com (J.B.); fbaeza2021@alu.uct.cl (F.B.); josefa.diaz2021@alu.uct.cl (J.D.); sfuentes2021@alu.uct.cl (S.F.); pletelier@uct.cl (P.L.); 2 Laboratorio Clínico, Hospital Dr. Hernán Henríquez Aravena, Temuco 4780000, Chile; felipegarrido.tecmed@gmail.com (F.G.); f.quinones2015@gmail.com (F.Q.)

**Keywords:** COVID-19, blood smear, hematology, laboratory medicine, biomarkers, inflammatory markers, NETs

## Abstract

*Background and Objectives:* Neutrophil extracellular traps (NETs) have been linked to hypercoagulability, immunothrombosis, and organ injury in COVID-19. Digital morphology of peripheral blood smears enables the identification of NET-compatible appearances (NET-like) in circulation, and associations between NET-like derived indices and clinical outcomes have been reported. However, evidence at hospital admission that relates smear NET-like burden to systemic inflammation and clinical severity remains limited. We therefore aimed to compare the burden of NET-like structures on admission smears according to disease severity and systemic inflammatory markers. *Materials and Methods:* We included 50 consecutively enrolled adults hospitalized for COVID-19; samples were obtained within 24 h of admission. Severity was defined by the World Health Organization Clinical Progression Scale and grouped as moderate or severe. C-reactive protein (CRP), ferritin, and complete blood counts were measured; the neutrophil-to-lymphocyte ratio (NLR) and platelet-to-lymphocyte ratio (PLR) were calculated. Digital morphology assessed 200 leukocytes per patient; the presence of morphological abnormalities, including NET-like events per patient, was recorded. We additionally quantified NET-like events per 100 white blood cells (NET-like/100 WBC) and the neutrophil extracellular trap–segmented neutrophil ratio (NNSR). *Results:* At admission, CRP, ferritin, NLR, and PLR of patients were above method-specific reference intervals. NET-like events were identified in 66% of patients. NET-like/100 WBC correlated positively with NLR (r = 0.312; *p* < 0.05). Patients with severe COVID-19 had higher NET-like/100 WBC than those with moderate disease (5.8 ± 7.34 vs. 14.14 ± 15.12; *p* = 0.0294). *Conclusions:* Digital morphological identification of NET-like structures on peripheral blood smears is frequent at admission and is associated with systemic inflammatory burden and with greater COVID-19 severity. These findings support the potential complementary value of reporting NET-like events for initial risk stratification in the clinical laboratory.

## 1. Introduction

Coronavirus disease 2019 (COVID-19) remains a global health problem, with ongoing outbreaks that can progress to severe disease (1). Several factors may account for this phenomenon, including vaccination coverage in the population. Reports from the World Health Organization (WHO) indicate that, at the global level, only 31% of the population is immunized [1]. In addition, the emergence of new SARS-CoV-2 variants and their rapid spread may lead to reinfections and immune escape, affecting susceptible patients [2].

A variety of hematological alterations in patients with severe COVID-19 have been demonstrated [3], which may constitute potential prognostic biomarkers of the disease [1,3,4]. Among these, the elevated markers of systemic inflammation, such as the neutrophil-to-lymphocyte ratio (NLR) and platelet-to-lymphocyte ratio (PLR), have been associated with severe clinical presentations [5,6]. Furthermore, abnormalities in peripheral blood cell morphology have been described [7,8]. Regarding inflammatory serum biomarkers, like C-reactive protein (CRP) in COVID-19 patients, it has been demonstrated that their increased concentration is related to leukocytosis and is unfavorable in this disease [9].

Neutrophils play a central role in responses to pathogens as part of innate immunity and can form Neutrophil Extracellular Traps (NETs), which can be identified by experimental methods [10,11]. NETs were first described by Brinkmann et al. (2004), who characterized them as extracellular fibrillar networks derived from decondensed chromatin expelled by activated neutrophils [12]. Also, the process of inducing NETs has been called NETosis [13]. In the classical lytic form of NET release, nuclear architecture and plasma-membrane integrity are lost; however, “vital” forms of release have also been reported, in which the neutrophil membrane remains intact. NET fibers are composed of DNA and histones and include granular proteins, including neutrophil elastase, cathepsin G, and myeloperoxidase [12]. NETs have also been associated with hypercoagulability, immunothrombosis, and organ injury in COVID-19 [14].

Following the structural characterization of NETs, translation into clinical practice has advanced through the use of digital morphology systems capable of recognizing NET-like morphological events in peripheral blood smears on the basis of decondensed chromatin with reticular projections [15,16]. According to Wang et al., in a study of patients with COVID-19, digital morphology analysis of peripheral blood smears represents a rapid and cost-effective approach with good diagnostic value for the assessment of this pathology. These studies have documented the frequency of NET-like events and have associated quantitative indices related to NET-like structures with mortality risk in COVID-19, enabling a quantitative characterization of NET-related changes in peripheral blood [17].

Most evidence on NETs in COVID-19 derives from plasma biomarkers; consequently, evidence linking the morphological quantification of NET-like structures in blood smears with systemic inflammatory parameters and clinical severity at hospital admission remains limited. Therefore, the aim of this study was to compare the NET-like burden in blood smears at admission according to severity and examine its relationship with systemic inflammatory parameters in patients with COVID-19.

## 2. Materials and Methods

### 2.1. Participants

The study design adopts a retrospective, single-center cohort with cross-sectional assessment at hospital admission. A total of 50 consecutive patients older than 18 years, admitted to Dr. Hernán Henríquez Aravena Hospital, Temuco, Chile, were included in the study. The study was approved by the Scientific Ethics Committee of the Araucanía Sur Health Service (No. 144/2020) and was carried out in accordance with the ethical requirements established in the Declaration of Helsinki. The patients were diagnosed with COVID-19 according to criteria established by the Chilean Ministry of Health. Briefly, the patients presented with an acute infection with two or more of the following signs and symptoms: fever, cough, respiratory distress, anosmia, myalgia, among others, in addition to confirmation by molecular detection of SARS-CoV-2 from nasopharyngeal and oropharyngeal swab specimens. Disease severity was defined according to the WHO Clinical Progression Scale [18].

### 2.2. Laboratory Analyses

All the samples were collected within the first 24 h of hospital admission. Measurements of inflammatory biomarkers CRP and ferritin were performed using the Cobas^®^ 8000 series modular analyzer (Roche Diagnostics, Mannheim, Germany), by immunoturbidimetric (CRP) and electrochemiluminescence (ferritin) assay. Complete blood count and differential leukocyte count were performed using a Sysmex XN-2000 hematology analyzer (Sysmex Corporation, Kobe, Japan); additionally, NLR and PLR were calculated by dividing the absolute neutrophil count by the lymphocyte count and the platelet count by the lymphocyte count, respectively. Peripheral blood smear preparation and May–Grünwald–Giemsa staining were performed using the automated Sysmex DI-60 (Sysmex Corporation, Kobe, Japan) within 120 min after blood collection. The reference intervals shown in Table 1 derive from the institutional, method-specific ranges in use at the Hospital Dr. Hernán Henríquez Aravena Clinical Laboratory during the study period and were used solely to contextualize values as within or outside reference limits.

Automated leukocyte differentiation was performed using the CellaVision^®^ DM1200 system (CellaVision AB, Lund, Sweden), configured to count 200 cells per patient (10,000 leukocyte images in total), with automated morphology pre-classification. The automated blood cell pre-classification and morphology description were reviewed and confirmed by two experienced cytomorphology professionals, following national recommendations. In the result analysis, patients with morphological abnormalities in greater than 5% of the evaluated cell line of peripheral blood cells were reported as positive for morphological abnormality according to clinical internal consensus in order to avoid sporadic findings of uncertain significance. For neutrophils, the morphological changes assessed included toxic granulation, hypersegmentation, hyposegmentation (referred to in text as “Pseudo–Pelger–Huët anomaly”), left shift, Döhle bodies/zones, abnormal nuclear projections (referred to in text as “satellite chromatin”), apoptotic cells, and ring neutrophils. In addition, NET-like events were identified as neutrophil nuclei without cytoplasm with polarized chromatin projections forming a spider-web-like reticular pattern. Their presence was recorded as positive in patients when they represented greater than 1% of evaluated leukocytes, because normal healthy people should not possess any type of Smudge cells, including NET-like events [16]. Based on these observations, one related NET-like parameter obtained was NET-like per 100 white blood cells (NET/100WBC), and the NET–segmented neutrophil ratio (NSNR) was calculated as described by Wang et al. [17]. Lymphocyte morphology variations included reactive lymphocytes, large granular lymphocytes, plasmacytoid cells, and cytoplasmic vacuolization. Monocyte morphological changes considered the presence of cytoplasmic vacuoles; while platelet evaluation included macroplatelets and vacuoles. RBC morphology changes included anisocytosis, polychromasia, basophilic stippling, and nucleated RBC.

### 2.3. Statistical Analysis

Statistical analyses were performed using GraphPad Prism version 6.0 (GraphPad Software, San Diego, CA, USA), using a *p*-value less than 0.05 (*p* < 0.05) as statistical significance. Continuous variables were expressed as means and standard deviations. For comparisons between two groups, Student’s *t* test was applied for normally distributed data, and the Mann–Whitney test was used for non-parametric distributions. Correlation analyses between inflammatory markers, hematological quantitative parameters, and morphological alterations were conducted using Pearson’s correlation. The risk of severe COVID-19 at hospital admission was assessed using contingency tables to compare different parameters between moderate and severe COVID-19 groups. Cut-off values (COVs) for each parameter were established based on the reference intervals described in Table 1. For NET-like parameters, COVs were determined using the Youden index. Briefly, a ROC curve analysis was performed, and the Youden index (J) was calculated as the maximum value of (Sensitivity + Specificity − 1) to identify the optimal cut-off value.

## 3. Results

Among the 50 patients included, the progression scale classified 15 patients as having moderate and 35 as having severe COVID-19. Table 1 summarizes the baseline characteristics and admission laboratory findings. When admission hematologic parameters were compared by sex, significant differences were observed in red cell indices (mean ± SD): RBC (men 4.209 ± 0.73 × 10^12^/L vs. women 3.794 ± 0.54 × 10^12^/L, *p* = 0.028), hemoglobin (men 127.5 ± 21.68 g/L vs. women 110.4 ± 16.67 g/L, *p* = 0.0025), hematocrit (men 0.3844 ± 0.06074 L/L vs. women 0.3389 ± 0.04664 L/L, *p* = 0.0023), MCH (men 30.41 ± 0.9791 pg vs. women 29.17 ± 1.948 pg, *p* = 0.0148), and RDW-CV (men 13.48 ± 0.8474 vs. women 14.36 ± 1.648, *p* = 0.048).

**Figure 1:**
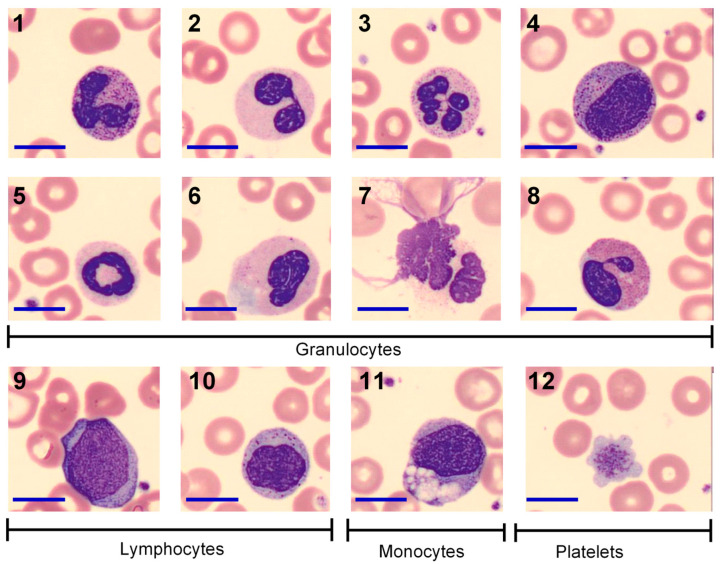
Abnormal blood cell morphology in COVID-19. 1–7 Abnormal morphology in granulocytes: 1 Neutrophil toxic granulation; 2 Neutrophil nuclear hyposegmentation; 3 Neutrophil nuclear hypersegmentation; 4 Immature granulocyte (left shift); 5 Ring Neutrophil; 6 Neutrophil Döhle body; 7 Neutrophil extracellular traps; 8 Eosinophil Irregular granule distribution; 9–10 Abnormal morphology in lymphocyte; 9 Reactive lymphocyte; 10 Large granular Lymphocyte; 11 Multivacuolated monocyte; 12 Macroplatelet. Scale bar = 10 μm.

Morphologic abnormalities considered to be positive are summarized in Table 2. The most frequent pathological neutrophil findings were toxic granulation (100%), Döhle bodies/zones (80%), nuclear hypersegmentation (74%), and satellite chromatin (62%) (Figure 1). Lymphoid abnormalities included reactive lymphocytes (60%), large granular lymphocytes (60%), and plasmacytoid lymphocytes (32%) (Figure 1). Macroplatelets were observed in 84% of patients. In the erythroid series, the most common findings were polychromasia (40%), anisocytosis (26%), and basophilic stippling (24%).

At admission, NLR and PLR were elevated relative to reference intervals, with no sex-based differences found (both *p* > 0.05). Serum inflammatory biomarkers CRP and ferritin were also increased in contrast to reference intervals, and ferritin levels were significantly higher in men than in women (men 1968 ± 1396 ng/mL vs. women 1205 ± 1114 ng/mL, *p* = 0.043). Also, ferritin correlated positively with hemoglobin (r = 0.516, *p* < 0.05), hematocrit (r = 0.460, *p* < 0.05), and MCHC (r = 0.653, *p* < 0.001), and inversely with RDW-CV (r = −0.526, *p* < 0.05). NLR correlated positively with the neutrophil absolute count (r = 0.828, *p* < 0.05) and inversely with lymphocyte (r = −0.994, *p* < 0.001) and monocyte absolute counts (r = −0.488, *p* < 0.001) (Table 3).

When inflammatory parameters were examined in relation to blood smear morphologic abnormalities, serum ferritin showed an inverse correlation with irregular eosinophil granule distribution (r = −0.419, *p* < 0.001). NLR correlated positively with the presence of satellite chromatin (r = 0.408, *p* < 0.001) and inversely with large granular lymphocytes (r = −0.397, *p* < 0.001). CRP correlated inversely with hypersegmented neutrophils (r = −0.377, *p* < 0.001) and with the presence of macroplatelets (r = −0.322, *p* < 0.001). For PLR, an inverse correlation was observed with ring neutrophils (r = −0.586, *p* < 0.001). No significant correlations were observed between NET-like metrics and CRP or ferritin (*p* > 0.05; Table 3).

NET-like events were identified on peripheral blood smears in 66% of hospitalized patients (Table 2). In addition, NLR showed a moderate positive correlation with the presence of NET-like events (r = 0.312, *p* < 0.05). Patients with severe COVID-19 exhibited a higher NET-like count per 100 white blood cells than those with moderate disease (14.14 ± 15.12 vs. 5.8 ± 7.341 NET-like/100 WBC; *p* = 0.0294), whereas NSNR did not differ significantly between moderate and severe cases (*p* = 0.066) (Table 4). Likewise, no significant correlations were observed between NET-like metrics and CRP or ferritin (*p* > 0.05).

For the risk analysis of severe COVID-19, the COVs determined via the Youden index were 7% for NET-like/100WBC and 8% for NSNR. The presence or absence of NET-like structures was also included in the analysis. Regarding single parameters, only PLR (OR = 4.23; 95%CI = 1.10–16.19; *p*= 0.04) and absolute lymphocyte count (OR = 4.57; 95%CI = 1.23–16.93; *p* = 0.04) were significantly associated with severe disease. Notably, the risk significantly increased in patients simultaneously presenting high ferritin levels and high NET-like/100WBC values (OR = 7.11; 95%CI = 1.23–40.98; *p* = 0.027). A combined elevation of PLR, absolute neutrophil count, and the presence of NET-like structures was also associated with increased risk (OR = 5.33; 95%CI = 1.27–22.32; *p* = 0.029).

## 4. Discussion

Although COVID-19 is no longer classified as a public health emergency and effective vaccination strategies are available, it remains a relevant health problem, with a proportion of patients progressing to severe disease. In this study, we characterized blood-smear morphological variations and NET-related parameters in hospitalized patients with COVID-19 and explored their relationships with systemic inflammatory markers.

With regard to inflammatory markers, ferritin, a key mediator within innate immune responses [5], was elevated in all patients and higher in men than in women, suggesting a more pronounced inflammatory status in males. Given ferritin’s role in inflammation, this finding may indicate that men are more predisposed to severe COVID-19 due to an exacerbated inflammatory response [19], supporting its potential as an indicator of poor clinical course in COVID-19 hospitalized patients. Consistently, ferritin correlated with erythrocyte indices, particularly MCH and MCHC, which is consistent with its fundamental role in iron metabolism and erythropoiesis [20]. In COVID-19, the overexpression of IL-1, IL-6, IFN-γ, and TNF-α has been implicated in perturbations of iron homeostasis and erythropoiesis [21].

NLR exhibited strong, expected correlations with relative neutrophil and lymphocyte counts. Neutrophilia has been linked to cytotoxic tissue injury [11]; a positive association with neutrophils and a negative association with lymphocytes reflect the differential immune response in inflammatory states, consistent with reports of NLR as a marker associated with severe COVID-19 [6,22] and as an accessible inflammatory biomarker. The reports of Letelier et al. [4] have shown significant relationships between NLR/PLR and severity; the elevation of both indices in our cohort reinforces that dysregulated inflammation is a key component of severe disease. Although sex-based differences in NLR/PLR were not significant, their overall elevation supports a state of immune hyperstimulation. In addition, prior work emphasizes a bidirectional interplay between the immune and coagulation systems [23].

The morphologic changes observed during COVID-19 indicate a complex, dynamic immune response, with the granulocytic lineage showing the most prominent alterations (toxic granulation, Döhle bodies, hypersegmentation, and left shift). Of note, we found a strong inverse correlation between PLR and ring neutrophils. In line with our observations, Jain et al. reported, in Intensive Care Unit (ICU) versus non-ICU patients, neutrophil abnormalities such as left shift, Döhle bodies, hypersegmentation, and the presence of ring neutrophils associated with severe COVID-19 [24].

In our cohort, NET-like events were detected on peripheral blood smears of hospitalized patients, in agreement with recent reports [17]. Moreover, patients with severe disease had a higher NET-like count per 100 WBC than those with moderate disease, whereas NSNR did not differ significantly across severity strata. In a cohort of 117 hospitalized patients, Wang et al. described higher NET-related measures in severe and deceased patients, with an increase in NSNR as well [17], and NET/100WBC rose substantially among non-survivors, performing as a mortality-risk indicator [17]. Although NETs are not routinely described in blood smear reports, our findings support the potential value of documenting NET-like events within hematology reports as a complementary element for severity assessment in COVID-19.

**Figure 2:**
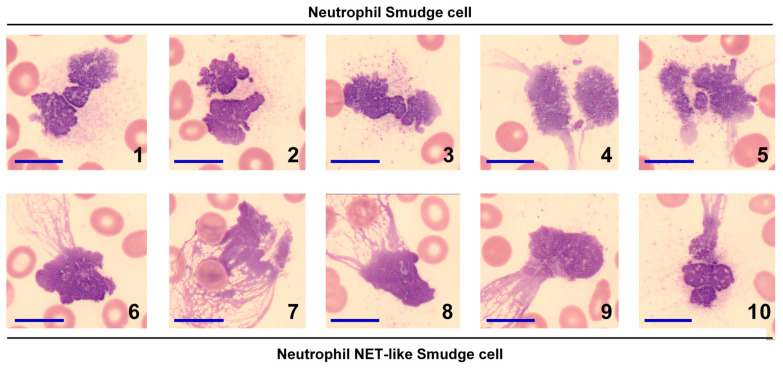
Morphological distinction between Neutrophil Smudge cells and NET-like Smudge cells. The upper panel (images 1–5) displays the classical morphology of Neutrophil Smudge cells, characterized by naked nuclei with surrounding granules but lacking cytoplasm. The lower panel (images 6–10) depicts Neutrophil NET-like Smudge cells, defined by naked nuclei with polarized, reticulated chromatin projections resembling a spider web. Notably, while cells 4 and 5 exhibit some nuclear projections, they lack the distinct polarized reticulation of NET-like forms and are therefore classified as standard Smudge cells. Abbreviation: NET = Neutrophil Extracellular Trap. Scale bar = 10 μm.

Importantly, by NET-like, we refer to morphological events compatible with NETs on blood smears, specifically the reticular, spider-web-like chromatin structures, which also are recognized by digital morphology analysis (Figure 2). However, smear microscopy cannot ascertain the biochemical composition (e.g., histones, neutrophil elastase, cathepsin G, myeloperoxidase) [25] nor the cell death pathway involved [13]; therefore, it does not, by itself, establish NETs in the compositional sense or NETosis as a mechanism. For this reason, we adopt the term NET-like throughout.

The inflammatory milieu in severe COVID-19 has been extensively described. Neutrophils are abundant within pulmonary infiltrates (interstitium and peribronchiolar regions) and may form NETs as part of the response to SARS-CoV-2 [26]. Evidence indicates that IL-8 is a principal inducer of NET-mediated cell death (NETosis), with a central role in leukocyte recruitment [27,28], This creates a feed-forward loop in which recruited leukocytes continue to produce IL-8 and other mediators, promoting NETosis and perpetuating inflammation.

Of particular interest, we observed a moderate positive correlation between NLR and NET-like burden, a relationship not previously reported in COVID-19 to our knowledge. NLR is a simple, inexpensive, and widely available biomarker originally proposed by Zahorec [29], reflecting the dynamic interplay between innate and adaptive immune responses across diseases [30]. Prior work in other conditions supports an association between systemic inflammatory surrogates and NET formation: Larco et al. reported, in consecutive patients with acute ischemic stroke, an association between higher NLR and greater NET content [31]; similarly, in a cohort of 214 patients with renal cell carcinoma and tumor thrombus, elevated NLR was associated with neutrophil NETosis [32]. Evidence indicates that systemic inflammatory indices such as CRP, D-dimer, and NLR track with COVID-19 severity over time [33]. In our cohort assessed at admission, CBC-derived markers were informative: elevated PLR and lymphopenia each corresponded to roughly a fourfold higher odds of severe disease. Risk discrimination improved when inflammatory load was paired with smear-based NET-like burden. Specifically, concurrent hyperferritinemia with a high NET-like load identified patients at approximately a sevenfold higher risk. Similarly, the simultaneous presence of high PLR, neutrophilia, and NET-like events identified a group at about a fivefold higher risk, consistent with regional reports linking WBC metrics, NLR, PLR, neutrophils, and D-dimer to severe outcomes [4]. A limitation of our analysis is that D-dimer was not systematically measured at admission, which precluded its inclusion alongside CRP and ferritin in our combinations.

An important strength of this study is the standardized, automated workflow for peripheral blood film morphology, encompassing smear preparation, May–Grünwald–Giemsa staining, and digital image acquisition with expert validation, which, together with the examination of 200 leukocytes per patient across 50 patients (10,000 leukocyte images in total) and parallel assessments of erythrocyte and platelet morphology, yields a robust and well characterized dataset. The main limitation is the sample size of hospitalized COVID-19 patients; thus, these findings require confirmation in larger cohorts. Since we assessed admission data only, longitudinal changes in NET-like parameters remain to be explored in future prospective studies with digital morphology. Also, morphology-based NET-like burden may assist in enriching clinical trials of NET-targeted interventions by identifying patients with heightened neutrophil-driven inflammation at admission.

## 5. Conclusions

We observed a high prevalence of NET-like events in hospitalized patients with COVID-19, which correlated positively with NLR. When NET-related metrics were examined, patients with severe COVID-19 had a higher NET-like/100 WBC than those with moderate disease. Finally, incorporating the description of NET-like events into hematology reports may contribute to initial severity stratification in COVID-19, particularly in combination with inflammatory markers.

## Figures and Tables

**Table 1:** Baseline characteristics and laboratory findings on admission to hospital in patients with COVID-19.

Variable	Reference Intervals	Total (n = 50) Mean ± SD	Men (n = 32) Mean ± SD	Women (n = 18) Mean ± SD	* p * Value
age, years		53.68 ± 14.65	54.19 ± 2.45	52.78 ± 3.84	0.75
RBC, ×10^12^/L	3.8–5.8	4.06 ± 0.69	4.20 ± 0.73	3.794 ± 0.54	0.028 *
WBC, ×10^9^/L	4.0–12.0	12.67 ± 4,16	12.96 ± 4.62	12.15 ± 3.24	0.92
Hemoglobin, g/L	140–175	121.40 ± 21.50	127.5 ± 21.68	110.4 ± 16.67	0.0025 *
Hematocrit, L/L	35.0–47.0	36.80 ± 5.98	38.44 ± 6.07	33.89 ± 4.66	0.0023 *
MCV, fL	82.0–95.0	90.86 ± 4.37	91.66 ± 3.66	89.45 ± 5.23	0.085
MCH, pg	25.0–32.0	29.96 ± 1.51	30.41 ± 0.97	29.17 ± 1.94	0.0148 *
MCHC, g/L	320–360	329.6 ± 9.03	331.30 ± 8.32	326.7 ± 9.70	0.1546
PLT, ×10^9^/L	150–450	290.7 ± 101.7	266.4 ± 92.18	333.9 ± 105.9	0.022 *
RDW-SD, fL	37–46	45.09 ± 3.67	44.6 ± 3.365	45.97 ± 4.10	0.2758
RDW-CV, %	11.5–14.5	13.80 ± 1.26	13.48 ± 0.84	14.36 ± 1.64	0.048 *
MPV, fL	7–12	9.95 ± 1.06	10.00 ± 0.18	9.872 ± 0.25	0.689
Neutrophils, ×10^9^/L	2–8.2	10.82 ± 3.89	11.06 ± 4.27	10.39 ± 3.14	0.9398
Lymphocytes, ×10^9^/L	0.84–4.2	0.92 ± 0.67	0.90 ± 0.12	0.94 ± 0.12	0.8478
Monocytes, ×10^9^/L	0.16–0.96	0.80 ± 0.49	0.87 ± 0.49	0.6667 ± 0.4851	0.2316
Eosinophils, ×10^9^/L	0.08–0.6	0.06 ± 0.23	0.06 ± 0.24	0.05 ± 0.23	0.99
Neutrophils, %	50–58	85.04 ± 7.77	89.24 ± 5.46	90.94 ± 5.23	0.1985
Lymphocytes, %	21–35	7.88 ± 4.78	5.27 ± 3.69	5.50 ± 3.66	0.63
Monocytes, %	2–8	0.98 ± 2.72	1.15 ± 2.01	0.72 ± 1.17	0.553
NLR	0.107–3.19	18.20 ± 18.87	20.58 ± 21.93	13.67 ± 9.45	0.2175
PLR	46.79–218.01	323.9 ± 231.5	339.1 ± 253.8	295 ± 178.9	0.8951
CRP, mg/L	≤5	90.41 ± 91.02	80.33 ± 86.27	108.3 ± 98.87	0.36
Ferritin, ng/mL	30–400	1663 ± 1328	1968 ± 1396	1205 ± 1114	0.043 *
NET/100WBC, %	Not defined	11.64 ± 13.75	10.56 ± 13.31	13.56 ± 14.69	0.2612
NSNR, %	Not defined	12.80 ± 15.08	11.79 ± 14.95	14.6 ± 15.56	0.2637

Abbreviations: RBC = Red blood cell count; WBC = White blood cell count; PLT = Platelets; MCV = Mean corpuscular volume; MCH = Mean corpuscular hemoglobin; MCHC = Mean corpuscular hemoglobin concentration; RDW-SD = Red Cell Distribution Width standard deviation; RDW-CV = Red Cell Distribution Width coefficient of variation; MPV = Mean platelet volume; NLR = Neutrophil-Lymphocyte Ratio; PLR = platelet-lymphocyte ratio; CRP = C reactive protein. NET/100WBC = Neutrophil extracellular Tap (NET) per 100 white blood cells; NSNR = NET-Segmented Neutrophils Ratio; SD = Standard deviation. *p*-value indicate differences between men and women. * *p* < 0.05.

**Table 2:** Peripheral blood smear morphologic findings in hospitalized patients with COVID-19.

Morphological Finding	Percent (%)	Morphological Finding	Percent (%)
Neutrophils		Lymphocytes	
Toxic granulation	100	Large granular lymphocytes	60
Pseudo–Pelger–Huët	34	Reactive lymphocyte	60
Nuclear hypersegmentation	74	Plasmacytoid lymphocytes	32
Döhle Bodies	80	Lymphocyte vacuolation	16
Satellite Chromatin	62	Erythrocytes	
Left Shift (PMC, MC, metaMC, Band)	58	Anisocytosis	26
Apoptotic nucleus	6	Polychromasia	40
NET-like	66	Basophilic stippling	24
Ring Neutrophils	4	Nucleated red blood cells	12
Eosinophils		Platelet	
Irregular granule distribution	26	Macroplatelets	84
Monocytes		Vacuolated platelets	8
Multivacuolated monocytes	86		

The table reports the proportion of patients who exhibited each finding. Positivity was defined as a frequency exceeding five percent of evaluated cells. For NET-like events, the metric was quantified, and presence was additionally recorded when at least one percent of leukocytes displayed the finding. Abbreviations: PMC = Promyelocyte; MC = Myelocyte; metaMC = metamyelocyte; Band = Band Neutrophil.

**Table 3:** Correlation coefficients (r) between inflammatory markers and hematologic parameters and blood smear morphology.

Marker	Edad	Sexo	HCT	HGB	MCH	MCHC	RDW-CV	Neu %	Lim %	Mon %	Eo %	ANC	ALC	NLR	PLR	IG %	N/IG R	Left Shift	Pseudo–Pelger–Huët	NET/100 White Blood Cells	NSNR	Hypersegmented Neutrophils	Satellite Chromatin	Ring Neutrophils	NET-Like	Large Granular Lymphocytes	Plasmocytoid Lymphocyte	Eosinophil Irregular Granule Distribution	Macro Platelet
CRP	0.149	−0.004	0.056	0.049	−0.038	−0.09	−0.038	0.073	−0.020	−0.123	−0.01	0.009	−0.358 *	0.014	−0.032	0.296 *	0.339 *	−0.166	0.125	−0.18	−0.1821	−0.377 *	−0.241	0.082	−0.149	0.053	0.288	−0.113	−0.322 *
Ferritin	−0.191	−0.402 *	0.460 *	0.516 *	0.675 **	0.653 **	−0.526 *	0.101	−0.081	0.127	−0.353	0.164	0.078	0.061	0.153	−0.029	0.026	0.260	0.138	0.301	0.323	−0.237	−0.087	−0.185	0.071	0.056	−0.014	−0.419 *	0.078
NLR	0.561 **	−0.191	0.002	0.015	0.168	0.172	−0.170	0.828 **	−0.994 **	−0.488 **	−0.496 *	0.448 *	−0.506 *	---	−0.286 *	0.102	−0.072	−0.118	−0.289 *	0.219	0.215	0.218	0.408 *	0.118	0.312 *	−0.397 *	−0.358	−0.311 *	0.269
PLR	−0.031	0.046	−0.017	−0.013	0.077	0.019	0.065	−0.121	0.322 *	−0.036	−0.129	0.108	−0.383 *	−0.286 *	---	0.177	−0.046	0.286 *	0.029	0.096	0.101	−0.022	−0.198	−0.586 **	−0.205	0.206	0.133	0.171	−0.001

Abbreviations: RBC = Red blood cell; HCT = Hematocrit; HGB = Hemoglobin; MCH = Mean corpuscular hemoglobin; MCHC = Mean corpuscular hemoglobin concentration; RDW-CV = RBC distribution width; Neu = Neutrophils; Lim = Lymphocytes; Mon = Monocytes; Eo = Eosinophils; Bas = Basophils; ANC = Absolute Neutrophil Count; ALC = Absolute Lymphocyte Count; NLR = Neutrophil-Lymphocyte Ratio; PLR = platelet-lymphocyte ratio; IG% = Immature Granulocyte; N/IG R = Neutrophils/immature granulocytes ratio; NET-like = Neutrophil Extracellular Traps-like; NSNR = NET-Segmented Neutrophil Ratio; * *p* < 0.05; ** *p* < 0.01.

**Table 4:** NET-like parameters according to disease severity in hospitalized COVID-19 patients.

Parameter	Moderate	Severe	p Value
NET/100 white blood cells	5.8 ± 7.341	14.14 ± 15.12	0.0294 *
NSNR	6.546 ± 8.404	15.48 ± 16.55	0.066

Abbreviations: NET = Neutrophil Extracellular Traps; NSNR = NET-Segmented Neutrophil Ratio. * *p* < 0.05.

## Data Availability

The data presented in this study are available on request from the corresponding author. The data are not publicly accessible due to privacy restrictions.
